# Design and Validation of Virtual Reality Task for Neuro-Rehabilitation of Distal Upper Extremities

**DOI:** 10.3390/ijerph19031442

**Published:** 2022-01-27

**Authors:** Debasish Nath, Neha Singh, Megha Saini, M. V. Padma Srivastava, Amit Mehndiratta

**Affiliations:** 1 Centre for Biomedical Engineering, Indian Institute of Technology Delhi (IITD), New Delhi 110016, India; bmz198302@cbme.iitd.ac.in (D.N.); nehasingh0407@gmail.com (N.S.); megha.saini306@gmail.com (M.S.); 2 Department of Neurology, All India Institute of Medical Sciences (AIIMS), New Delhi 110029, India; vasanthapadma123@gmail.com; 3 Department of Biomedical Engineering, All India Institute of Medical Sciences (AIIMS), New Delhi 110029, India

**Keywords:** virtual reality, stroke, neuro-rehabilitation, distal upper extremities, performance metrics

## Abstract

Stroke, affecting approximately 15 million people worldwide, has long been a global cause of death and disability. Virtual Reality (VR) has shown its potential as an assistive tool for post-stroke rehabilitation. The objective of this pilot study was to define the task-specific performance metrics of VR tasks to assess the performance level of healthy subjects and patients quantitatively and to obtain their feedback for improving the developed framework. A pilot prospective study was designed. We tested the designed VR tasks on forty healthy right-handed subjects to evaluate its potential. Qualitative trajectory plots and three quantitative performance metrics—time taken to complete the task, percentage relative error, and trajectory smoothness—were computed from the recorded data of forty healthy subjects. Two patients with stroke were also enrolled to compare their performance with healthy subjects. Each participant received one VR session of 90 min. No adverse effects were noticed throughout the study. Performance metrics obtained from healthy subjects were used as a reference for patients. Relatively higher values of task completion time and trajectory smoothness and lower values of relative % error was observed for the affected hands w.r.t the unaffected hands of both the patients. For the unaffected hands of both the patients, the performance levels were found objectively closer to that of healthy subjects. A library of VR tasks for wrist and fingers were designed, and task-specific performance metrics were defined in this study. The evaluation of the VR exercises using these performance metrics will help the clinicians to assess the patient’s progress quantitatively and to design the rehabilitation framework for a future clinical study.

## 1. Introduction

Stroke, either an ischemic or a hemorrhagic, is a global medical emergency and one of the leading causes of high morbidity and mortality [1]. According to WHO, approximately 15 million people suffer a stroke annually, out of which 5 million are left permanently disabled [1]. Depending upon the location and severity of brain lesions, stroke survivors suffer from functional impairment associated with sensory, motor, and cognition. Residual upper limb disabilities persist in the chronic phase of stroke among approximately 66% of the stroke survivors [2]. Post-stroke rehabilitation is recommended to overcome persistent disabilities and to be able to independently perform the Activities of Daily Living (ADL). Conventionally-recommended rehabilitation therapy is usually time-consuming, labor-intensive, and lacks a naturalistic approach [3]. Lack of quantitative assessment and high clinical load results in therapist burnout and limits the effectiveness of traditional rehabilitation-methodologies. Furthermore, performing high repetitions with no quantifiable performance and feedback over a period may be monotonous and can reduce the patient’s active engagement in the training sessions [4]. Virtual reality (VR)-based rehabilitation methods have shown some promising results [5]. Nevertheless, most of the Virtual Reality studies emphasized recovery of proximal upper extremities [6], with limited focus on distal extremities, which play a vital role in performing ADL and require more intensive rehabilitation due to the requirement of fine optimal control at distal joints [7], and, therefore, are strongly associated with the quality of life [8]. Hence, targeting distal joint rehabilitation is essential for patients with stroke to facilitate the transfer of clinical gains to ADL. Therefore, an approach facilitating quantitative assessment of the performance, acting as an interesting and encouraging medium, providing adherence, and focusing on distal upper limb rehabilitation, is urgently needed.

Virtual Reality technology, comprising interactive simulated environments to enhance the user’s motivation through appropriate performance-derived feedback (visual, auditory, or haptic) provides a similar to real-world experience. Such experience can motivate the user to engage intensively for a long time without any boredom, which is crucial for a successful rehabilitation regime [2,3]. One important aspect of VR is its naturalistic approach, through which VR is able to transition the user into the virtual world and respond in real-time to the body’s movement in a naturalistic manner [4]. The ecological validity associated with VR-based rehabilitation has yet to be fully established [5]. However, as per available literature, depending upon the VR interface used for visualization and interaction, VR permits a naturalistic sensory-motor interaction between the user and the created virtual world [4,6]. Importantly, by establishing such immersiveness, VR can provide the user with an ecologically valid platform. VR therapy can be administered through an immersive (using a head-mounted display device) or non-immersive (without using any head-mounted display devices) approach. Literature has reported the use of the non-immersive VR approach to be a more viable solution as compared to immersive VR in terms of reduced cost (in limited economic settings), the ability to be integrated with various easily accessible interfaces [7,8,9], and having a lower chance of virtual motion sickness when used for a prolonged period [10,11]. VR has emerged as a complementary approach to increase therapeutic effectiveness addressing a few limitations associated with conventional rehabilitation [9,10], thereby, encouraging higher repetitions and exercise adherence [11,12]. It can facilitate the objective assessment of treatment response and progress throughout the intervention in addition to sharing the clinical load. VR-based therapy has shown its clinical potential for improving upper limb motor function [13,14,15] and different domains of cognition [16,17,18] when compared with an intensity-matched traditional rehabilitation [19]. However, most of the commercially-used VR tools are not tailored to the individual rehabilitation requirements of the patients and lack scalability [20]. The effect of virtual reality-based rehabilitation has been studied among different cohorts with different stages of stroke recovery, i.e., acute [21,22], sub-acute [23,24,25], and chronic [14,25,26]. Yet, the optimal therapeutic effect of VR is still indecisive, owing to the lack of an optimal control group [21,22], limited sample size, lack of standardized protocol, variations in the study designs, and, very importantly, lack of quantifiable metrics to evaluate its effectiveness.

One of the key advantages of VR-based intervention is the assessment of patients’ performance can be objective and quantified through task-related metrics, benefitting both the therapist and the patients, which traditional rehabilitation lacks. Such metrics providing the information about the task performance could be monitored for the purpose of clinical evaluation, appropriate modifications of VR tasks, tailoring task difficulty levels as per individual requirement, and can further monitor the response to intervention even from a remote location. Though various VR tasks are reported in the literature to provide a suitable interactive, engaging, challenging, and customizable environment where a user can practice precisely and repetitively and be guided through appropriate feedback [27,28,29,30], task-specific performance metrics are not focused on much and need to be investigated further. Through our current research work, such task-specific quantitative performance metrics are to be defined and validated, with both the healthy subjects and stroke patients.

The primary objective of this work is to define the key performance metrics related to the designed joystick-based non-immersive VR rehabilitation to assess the performance of healthy subjects and patients with stroke. The performance metrics of healthy subjects for distal joints are to be used as a reference to compare and differentiate it with the performance of patients with stroke in clinical settings. Furthermore, the subjective feedback and the learnings obtained from the healthy subjects and patients will be helpful in establishing the protocol and rehabilitation framework dedicated to patients with stroke.

## 2. Materials and Methods

### 2.1. Subjects

A pilot prospective interventional study in clinical settings was designed. The Institutional Review Board (IRB) at All India Institute of Medical Science (AIIMS), New Delhi, India, approved the study under protocol-number IEC-229/11.4.2020. A signed consent form was obtained from all the participants before enrollment in the study. Right-handed healthy subjects from our institute (*n* = 40; Male/Female = 35/5; Age-(Mean ± SD= 34.45 ± 10.97 years) with inclusion criteria—age 18–70 years, having no neurological disorder/hypertension/diabetes, volunteered for this study. Two right-handed patients (*n* = 2), with stroke, demographic information presented in Table 1, were enrolled having inclusion criteria, age 18–70 years, having ischemic/hemorrhagic stroke, Mini-Mental Scale (MMS) = 24–30, and Modified Ashworth Scale (MAS) = 1, 1+, 2. Apart from the demographic details, Fugl-Meyer Assessments of Upper Extremities (FMA-UE) were also obtained, as presented in Table 1.

**Figure 1:**
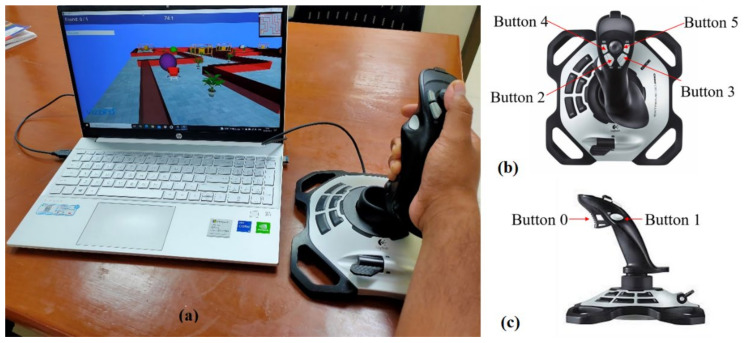
(**a**) VR setup with joystick and the subject performing the tasks, (**b**) top view of the joystick, and (**c**) side view of the joystick.

During the execution of the process, subjects’ forearms were kept fixed with the help of pillow support while performing the tasks to avoid any compensatory movements from proximal muscles/joints and to ensure the use of only distal upper extremities. A representative subject performing the tasks with the VR setup is shown in Figure 1a. First, the patients were asked to complete the tasks with their unaffected hand and then, later, with the affected hand. Appropriate rest-time of around 2 min was given to both the healthy subjects and patients in between different tasks to avoid any fatigue. The data acquisition process was performed under the supervision of an experienced physiotherapist present throughout the session. 

A subjective questionnaire form (SQF), specifically designed for this study, was administered to record healthy subjects’ and patients’ experiences regarding the usability of the joystick and VR tasks (Supplementary Table S1). The SQF focused on the subject’s interestingness and controllability of the VR tasks, user-friendliness, pain/fatigue/ adverse event experienced, individual suggestions, etc. The SQF has two parts: part A is common to both healthy subjects and patients; part B is specific to patients.

### 2.2. Hardware

Virtual environments, consisting of a sequence of VR-tasks, were developed using a Python-based software named ‘Vizard’ (^®^WorldViz LLC, Santa Barbara, CA, USA). A joystick from Logitech (Logitech Extreme 3d Pro, Lausanne, Switzerland) was used as an interface for the participant and the virtual environments. Figure 1b,c show the top and side views of the joystick used in the study, respectively. The yaw and pitch angles of the joystick device are ±15 degrees on each side from its mean position. Yaw motion of joystick was used for wrist flexion/extension, while pitch angle of the device helps in performing wrist radial/ulnar deviation. The device has six switches that can be used to train finger movements during the tasks. Button ‘0’ is used for index finger flexion and extension, and the other buttons are used for thumb abduction and adduction. These available motions of the joystick are useful for executing and learning the most useful and functional movements required for independently performing ADL.

### 2.3. Virtual Environment

**Figure 2:**
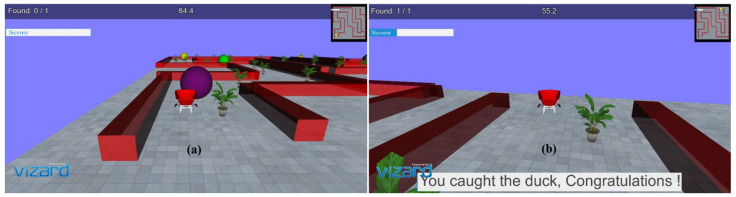
(**a**) Obstacles and task environment; (**b**) GUI and visual feedback on success.

In the virtual environments developed, a stimulus (3D duck model) is presented as a target to reach within a specified time limit. Different predefined 3D models in the form of obstacles are present on the driving path. It focuses on the critical consideration of the movements required for rehabilitation of distal joints of patients with stroke with the intended movements similar to the movements used in ADL, i.e., as wrist flexion/extension, radial and ulnar deviation, index finger flexion/extension, and thumb abduction/adduction. In each task, the participant was required to reach the pre-defined target position within a given time limit, avoiding collision with the obstacles. The movement, i.e., driving a car, allows the subjects to familiarize themselves with the 3D environments and motivates them to adhere to the tasks. The movements performed through the joystick are projected into the 3D environments, which causes a virtual motion of the 3D car object present in the 3D world. Colliding with obstacles creates an error, and the participant gets an error notification through audio feedback. Yaw movements of the joystick drive the car forward or backward, corresponding to wrist radial/ulnar deviation, and pitch angle turns the car left or right corresponding to wrist flexion/extension; the movements are importantly used in ADL. Each of these movement tasks was provided with multiple levels to make the environment more engaging, challenging, and encouraging for the patients for better compliance. One 90-min session of VR tasks was given to each of the healthy subjects and the patients. All of them were asked to sit comfortably on a chair with their back straight and place their arm on the table with the elbow ~120 degrees. All subjects were instructed to use and control the joystick using their wrist and fingers with limited use of their proximal parts. Before starting the VR task, all subjects were given one practice session of 5–10 min to make themselves comfortable with handling the joystick and to familiarize themselves with virtual environment setup. All the relevant instructions regarding the VR task were made visible to the participants before starting each task. At the end of each level, the performance level is displayed quantitatively (score) and qualitatively (progress bar). Appropriate visuals as per task performance, such as “You have done well! Congratulations” or “Try better next time! Please do well”, are displayed to motivate the user at this point. Figure 2a,b show the snapshots of the virtual environment and GUI designed at various time points of task execution. The developed tasks provided a desktop-based solution and did not incorporate any large screen or head-mounted device (HMD), which could affect the presence and immersion level of the user.

### 2.4. Task Description

**Figure 3:**
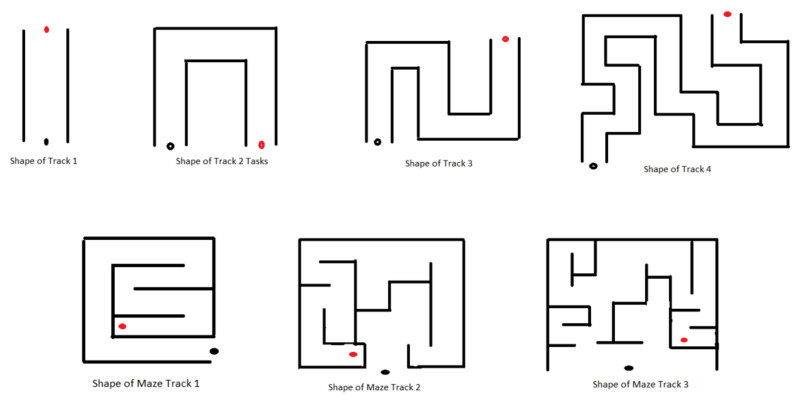
Representative shapes of various tracks used in designing VR tasks; black dot: starting position of the car; red dot: target position of the duck.

Two types of task environments were created:

i. **Individual Environments (IE):** IE tasks aim to introduce and make the subjects familiar with the different factors, such as time limits, the number of obstacles, various shapes of 3D tracks designed (Figure 3), etc., each factor corresponding to each task with difficulty level increased within the task order to keep the patients motivated. The details of the difficulties in IE are described in Table 2. Increasing the number of obstacles on the track can add complexity as it will compel the subject to make wrist joint movements avoiding a collision. The intent behind increasing such complexity is to compel the subject to make more precise and specific movements and, hence, to provide more intensive practice (wrist flexion/extension, ulnar/radial deviation). Furthermore, such a gradual increase in task complexity will keep the subject involved and encouraged throughout the session. Similarly, making the movements time-bounded can add a difficult element to the task environment as it requires more concentration, alertness, and planning to complete the task successfully. Various shapes of the tracks (Track 2, 3, and 4) were designed to present different environments, including the difficulty elements described above, which will keep the subjects involved throughout the process. In addition, these tracks are designed with a gradual increase in track length to provide more time to practice the movements more intensively (wrist ulnar and radial deviations). Tasks involving button pressing actions by using a thumb or index finger are advanced features that are incorporated as difficulty factors (in Module 6: Level 1 to Level 6). Individual maze tracks requiring planning execution and thinking capability were designed in the most advanced feature in the task levels of module 7.
ii. **Combined Environments (CE):** CE tasks were developed over the exact shapes of the 3D tracks (Figure 3) used in IE but combined with multiple factors of difficulties (described above) simultaneously to make the tasks more challenging and permit the subjects to use the experience gained from IE tasks. The details of difficulties in CE are described in Table 3. The subjects were assigned the CE tasks only after successful completion of the IE tasks.

### 2.5. Tasks

All the designed tasks can be divided into two groups in the aspect of targeted rehabilitation:

i. **Motor tasks:** Motor tasks require wrist and finger (thumb and index) movements to reach the target, while avoiding obstacles in the 3D world. Tasks of IE modules 1–5 (Table 2) focus on motor tasks.
ii. **Motor + Cognitive tasks:** These tasks require motor execution, memory, and planning capability. Tasks of IE modules 6–7 focus both on motor and cognition. Tasks of IE module-6 require the subject to remember a particular sequence of buttons instructed before task initialization and to press the joystick buttons in the exact sequence to remove the obstacles and move forward, reaching the target. Pressing an incorrect key was recorded as an error. Three different maze tracks were designed in IE module 7 tasks, for which the subject has to decide the correct path by viewing the top view of the 3D world to reach the target.

### 2.6. Outcome Performance Measures

The following three performance outcome measures are computed from the recorded data:

i. **Time taken to complete the task (TCT):** Time taken to complete the task is defined by:

(1) $$\mathrm{TCT}\;=\frac{Time\;taken\;to\;complete\;the\;task}{Specified\;time\;limit\;for\;the\;task}\times 100\;\left(\%\right)$$

The parameter TCT was used to examine whether the subject could complete a specific task within a reasonable time duration. As per our prior experience with the patients and available literature, a maximum of 90 min of a session is enough for the patient [12,13,14]. Therefore, taking care of this total time duration, an appropriate and reasonable time limit was set for each particular task level in order to complete the task. Values of TCT less than 100% indicate that the subject was able to complete a particular task successfully within a given time limit. Values of TCT more than or equal to 100% indicates that the subject was slow to achieve the target within the specified time. A decrease in TCT over the sessions is a success indicator that will indicate better learning and execution of tasks.

ii. **Relative percentage error**: Relative percentage error is defined by:

(2) $$\mathrm{Relative}\;\mathrm{percentage}\;\mathrm{error}=\frac{Distance\;covered\;by\;the\;subject-Ideal\;distance\;of\;track}{Ideal\;distance\;of\;track}\;\times 100(\%)$$

The relative % error parameter gives a numerical representation of the distance covered by the subjects to reach the target from the initial position. High values of % relative error indicate that the subject has traveled more distance than the ideal distance of the track designed, and vice-versa. This might be due to the tendency of the subject to deviate from the ideal path because of poor precision to control the car or off shooting the track. A reduction in relative percentage error during the session will indicate better control of motor control and coordination.

iii. **Smoothness of trajectory:** Smoothness of trajectory is defined by:

(3) $$\mathrm{Smoothness}\;\mathrm{of}\;\mathrm{trajectory}=\sqrt{\frac{1}{2}\int {\left((\frac{d^{2}x}{dt^{2}})\right)}^{2}+(\frac{d^{2}y}{dt^{2}}){)}^{2}+\left(\frac{d^{2}z}{dt^{2}}){)}^{2}\;dt\right)\frac{t^{5}}{s^{2}}}$$

where ‘*x*’, ‘*y*’, ‘*z*’ represents the 3D coordinates of trajectory path, ‘*t*’ is the motion time, and, ‘*s*’ is the motion distance. The lower the value of this parameter, the smoother is the motion trajectory, and the shorter will be the motion time, indicating improvement in the subject’s ability to control and coordinate the 3D motion.

iv. **Trajectory plots:** These are the plots of coordinates of motion path obtained at an interval of one second. Trajectory plots represent the variation in the shape of the path traveled for a particular duration of time. It can indicate the task performance by providing the path traveled by the subject for a particular task level and can be used to analyze whether the subject was able to complete the track or if any wrong trajectory was followed [15].

## 3. Results

All the healthy subjects (*n* = 40) and patients (*n* = 2) tolerated one 90-min session of VR task without any complaints. The time taken to complete the tasks, 3D coordinates of movements of the virtual object, and the number of errors in each task level was recorded for every individual throughout the sessions for further analysis. The three outcome measures described above were computed from the recorded data. Track-wise results of both IE and CE VR tasks were calculated. The results of a representative track (Track 4) are described in detail below for healthy subjects and patients. Results from other tracks are provided in detail in Supplementary Figures S1–S6. For patients’ affected hands, data were acquired for both the time-bound and no time-bound scenarios. Time-bound data was helpful in comparison against healthy subjects. The same VR tasks with no time-bound are employed for a better analysis, allowing the patients to complete the tasks at their ease.

### 3.1. Time Taken to Complete the Task (TCT)

During the 90-min session, the mean TCT values of the healthy subjects increased relatively by 8.12% from IE-L1 to IE-L2 of the representative track 4 (Table 4). Similarly, a relative increase of 34.9% TCT was observed from CE-L1 to CE-L15 for track 4. However, a relative decrease in the values of mean TCT has been observed from CE-L2 to CE-L3, CE-L10, to CE-L11, and CE-L13 to CE-L14, respectively. 

**Figure 4:**
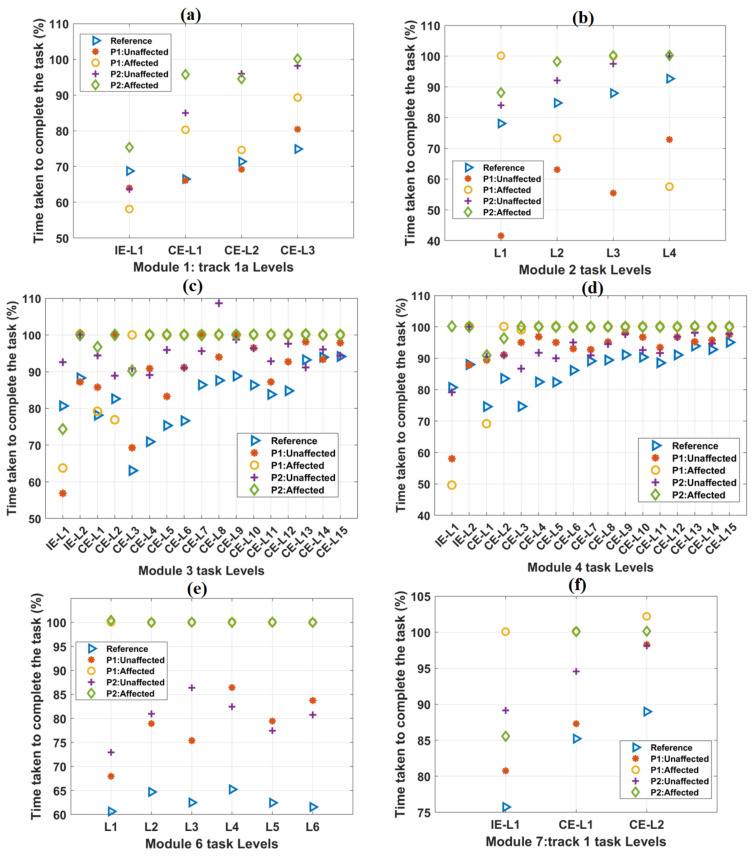
The variation of the TCT parameter with the increase in difficulty levels in (**a**) Module 1, (**b**) Module 2, (**c**) Module 3, (**d**) Module 4, (**e**) Module 6, and (**f**) Module 7 tasks. In each case, the scores of both the patients were plotted against the mean score obtained from forty healthy subjects, which was taken as a reference. In most of the cases, TCT scores obtained from the affected hands of both the patients were equal to 100%.

Similarly, the unaffected hands of both the patients showed an increasing trend of TCT for IE and CE tasks of track 4 (Table 4). For the affected hands of both the patients with time-bound included, a relative increase in TCT was observed in IE task levels. However, from CE-L3 to CE-L15, both the patients showed TCT values equal to 100% (Table 4). For TCT values obtained from both the patients’ unaffected hands without time-bound, relatively increased trends were observed for IE and CE tasks of track 4. However, from CE-L3 to CE-L15, both the patients showed TCT values equal to greater than 100% (Table 4). The TCT values of the healthy subjects and the patients for other tracks were observed to be relatively similar to these results (Figure 4). The detailed results for other tracks were shown in Supplementary Figure S1.

The variations in TCT values with task difficulty levels in other tracks are shown in Figure 4. The mean TCT values for healthy subjects were computed and taken as a reference. As task difficulty increased, TCT values were observed to be increasing, except task levels in module 6 (Figure 4e). For module 6 task levels, TCT values were found to be almost equal. For all the IE and CE task levels, the reference TCT values were found to be less than 100%. In most of the task levels, TCT values were found to be 100% for the affected hands of both the patients. TCT values obtained from the unaffected hands of both the patients were found to be numerically closer to the reference values (Supplementary Figure S1). Without any time-limit, TCT values exceeded the maximum value of 100% (Supplementary Figure S1). 

### 3.2. Smoothness of Trajectory

The mean smoothness values of the healthy subjects increased relatively by 19.92% from IE-L1 to IE-L2 of the representative track 4 (Table 5) during the task sessions. Similarly, a relative increase of 54.28% smoothness was observed from CE-L1 to CE-L15 for track 4. Moreover, the unaffected hands of both the patients showed an increasing trend of smoothness for IE and CE tasks of track 4 (Table 5).

**Figure 5:**
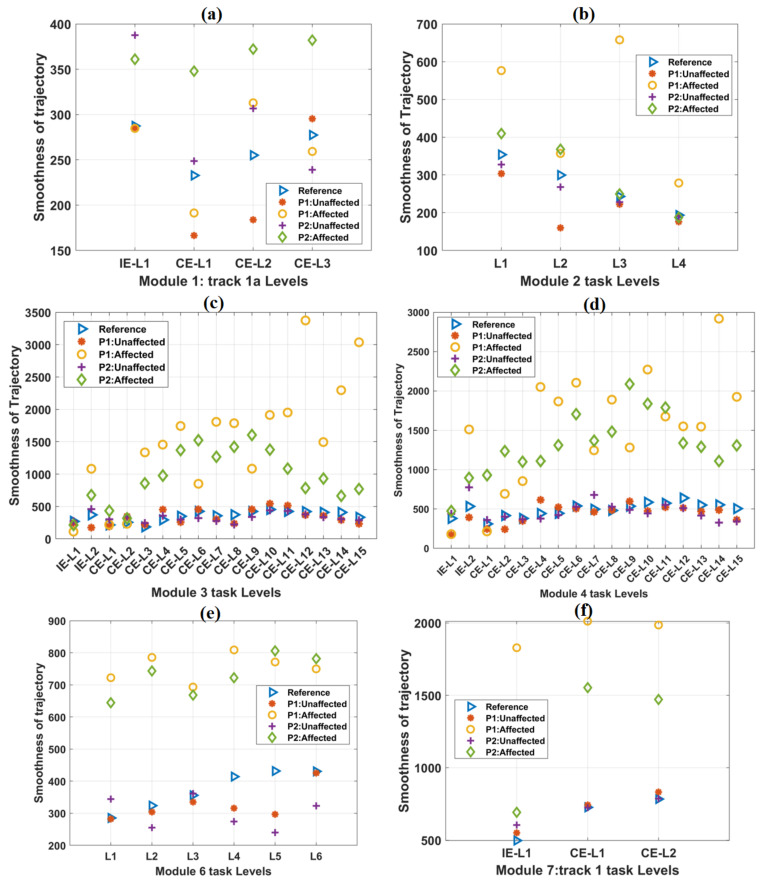
The variation of the Trajectory smoothness parameter with an increase in difficulty levels in (**a**) Module 1, (**b**) Module 2, (**c**) Module 3, (**d**) Module 4, (**e**) Module 6, and (**f**) Module 7 tasks. In each case, the scores of both the patients were plotted against the mean score of forty healthy subjects, which was taken as a reference. In most of the cases, trajectory smoothness values obtained from the affected hands of both the patients were relatively greater than these reference values.

For the affected hands of both the patients with the time-bound included, a relative increase in smoothness was observed in IE and CE task levels of track 4 (Table 5). For smoothness values obtained from both the patients’ unaffected hands without any time-bound, relatively increased trends were observed for IE and CE tasks of track 4 (Table 5). The trajectory smoothness values of the healthy subjects and the patients for other tracks were found to be relatively similar to these results (Figure 5). The detailed results for other tracks were shown in Supplementary Figure S3.

### 3.3. Relative % Error

**Figure 6:**
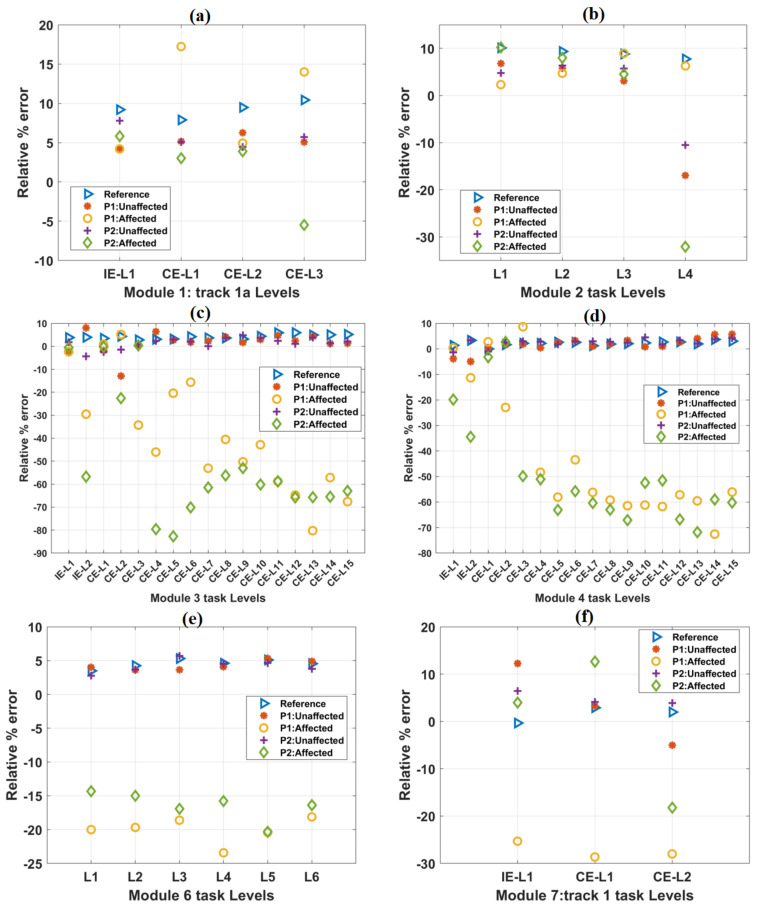
The variation of the relative % error parameter with the increase in difficulty levels in (**a**) Module 1, (**b**) Module 2, (**c**) Module 3, (**d**) Module 4, (**e**) Module 6, and (**f**) Module 7 tasks. In each case, the scores of both the patients were plotted against the mean score of forty healthy subjects, which is taken as a reference. In most of the cases, relative % error values obtained from the affected hands of both the patients were relatively less than these reference values.

The healthy participants’ mean percentage relative error values increased relatively in IE and CE task levels of track 4 (Table 6). Similarly, relatively increasing values of relative % errors were observed while the patients performed the IE tasks using their unaffected hands (Table 6). However, relatively decreasing values of relative % errors were observed while the patients were performing the IE tasks using their affected hands with a time-bound given (Table 6). Relatively decreasing values of relative % errors were observed while the patients performed the IE tasks using their affected hands without specifying time-bound (Table 6). The relative % error values of the healthy subjects and the patients for all other tracks were found to be relatively similar to these results (Figure 6). The detailed results for other tracks are shown in Supplementary Figure S5.

The variations in relative % error values with progress in task difficulty levels in other tracks are shown in Figure 6. Mean relative % error values for healthy subjects were taken as a reference. For module 2 task levels, a constant decrease in trajectory smoothness values was observed (Figure 6b). For module 6 task levels, these values were found to be in close range (e.g., L2: 4.2%, L3: 5.3%, and L4: 4.6%) with progress in task difficulty levels (Figure 6e). For the affected hands of both the patients in most of the task levels, relative % error values are found to be considerably less w.r.t the reference values obtained from healthy subjects. However, for time-unbound conditions, these values were found to be identical with the reference values (Supplementary Figure S3). Relative % error values obtained from the unaffected hands of the patients were numerically closer to the reference values (Supplementary Figure S3).

### 3.4. Trajectory Plots

**Figure 7:**
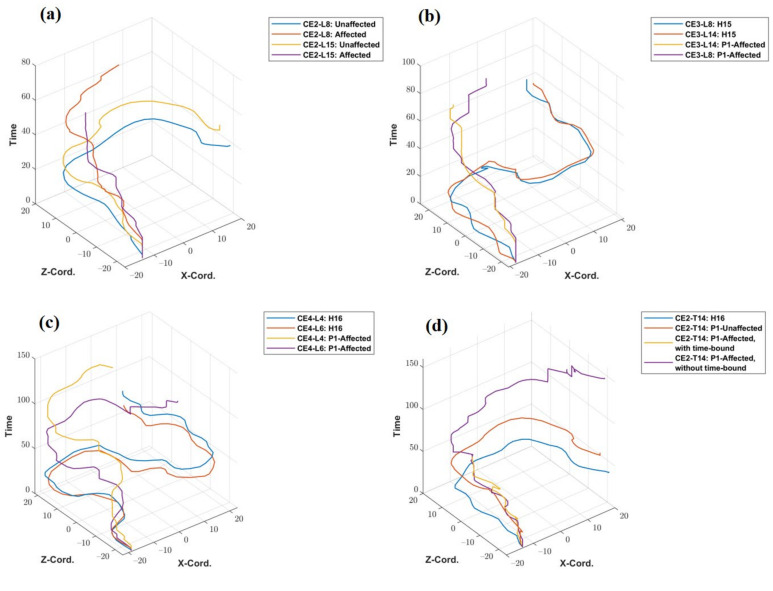
Trajectory plotted (**a**) for P1’s unaffected and affected hands for task levels CE2-L8 and L15, (**b**) for a healthy subject and P1’s affected hand for task levels CE3-L8 and L14, (**c**) for a healthy person and P1’s affected hand for task levels CE4-L4, and L6, (**d**) for a healthy subject and P1’s affected hand, with and without the time-bound conditions for task level CE2-L14.

The trajectory plots of the unaffected and affected hands of patient P1 for tasks CE2- L8 and L15 are shown in Figure 7a. The *x*- and *y*-axis of the trajectory plots represent the recorded X and Z—coordinates of the traveled path. The Y coordinate values remain fixed throughout the trajectory (In the default coordinate system of the Vizard software environment, the Y coordinate value represents height.). The *z*-axis of the plot represents the sampling time of the X and Z coordinates of the trajectory. The patient P1 took 42.27 s to complete CE2-L8 with his unaffected hand but failed to reach the target point even after utilizing the full 45 s of the specified time limit. Hence, the line showing the trajectory of the patient’s unaffected hand is incomplete and has a higher value in the time axis. Similarly, for CE3 (L8 and L14) and CE4 (L4 and L6), the trajectories of healthy subjects and that of the patient’s affected hand are plotted and shown in Figure 7b,c. Figure 7d shows the comparison of the trajectory of a healthy subject, the unaffected hand of the patient, and the affected hand of the patient (with and without specified time constraints). The patient could not complete the task using his affected hand within the time limit of 45 s, but when asked to perform without any time-bound, it took 236.23 s to reach the target. A plot showing trajectories of four representative healthy subjects (H5, H11, H14 and H20) and two stroke patients for the task-level CE3-L11 is given in Supplementary Figure S7. 

### 3.5. Subjective Questionnaire Feedback (SQF)

To obtain the subjective experience regarding the VR session, a subjective questionnaire feedback (SQF) has been designed specific to this study (Supplementary Table S1). All the healthy subjects and the patients found the entire VR setup safe and easy to use, and even recommended it for use at home, provided with the details of instructions and proper demonstration before using the system. All of them found the VR tasks easily understandable, enjoyable, motivating, and exciting to perform. Suggestions obtained from both healthy subjects and patients were noted, focusing on how to improve and customize this VR setup further (Supplementary Table S1).

According to the SQF, thirty-five percent of the healthy subjects had experienced visual discomfort and headache with pain in the shoulder after repeatedly performing the tasks (Q.12 of SQF part A: Supplementary Table S1). All of them had felt that the CE tasks were challenging to perform as compared to IE tasks (Q.15 of SQF part A: Supplementary Table S1). Cognitive load was especially experienced during the later tasks of CE, where the time-bound was less, and the number of obstacles was comparatively high. 

Both the patients were able to understand the instructions on how to handle the joystick and execute the tasks. The task GUI and audio-visual feedback of VR environments were interesting as per the patients. Having normal cognition levels (MMSE = 30), both were able to understand the displayed instructions and perform the tasks accordingly. Both of them were willing to use the entire setup at-home settings. However, for regular practice, they suggested changing the VR task elements, such as 3D models, including different types of obstacles and audio-visual cues at different time intervals, to keep their engagement high. Both patients recommended altering the button placements of the joystick for more comfortable use. P1 suggested increasing the initial width of track 3, 4, and maze tracks and gradually decreasing them to make the tasks more challenging. P1 also proposed to increase the GUI size of the top-view window to have better visibility. P1 has experienced visual discomfort due to the longer duration of VR tasks, as found in response to Q.12 of SQF part A (Supplementary Table S1). 

## 4. Discussion

A customized VR-based platform with multiple levels of training for motor rehabilitation of wrist and finger was designed, with quantifiable parameters to evaluate the progress of patients. The results demonstrated the variations of three objective quantitative parameters and qualitative trajectory plots obtained from the healthy subjects and two patients (for both unaffected and affected hands). These parameters can be used to describe a subject’s performance quantitatively at a specific task level. The primary goal of this study was to define the performance metrics to be used for comparing and differentiating the performance of a healthy individual and patients with stroke. The mean values of performance measurements serve as a benchmark against which the patient’s results can be compared. The unaffected hands of the patients had quite identical outcome measures, the same as the performance metrics of healthy subjects. The performance metrics of both the patients’ affected hands differed from the reference values obtained from the healthy subjects. 

### 4.1. TCT

As the subjects were exposed to the environment for the first time, the increase in task difficulty level caused an increased TCT in IE and CE task levels. It is possible that the reduced TCT values in some of the adjoining levels of CE tasks were due to the familiarity of the subjects with the task contents. For the unaffected hands of both the patients, TCT values were observed to be identical to the values of the reference values. In some of the tasks, the patients achieved the goal earlier with their affected hands than the standard reference, but with a high number of errors (for Module 2-L2: Reference= 84.7%, P1 = 63.1%, number of collisions with obstacles = 6). However, the TCT values become 100% when the patients could not reach the target with their affected hands, even after fully utilizing the specified time limit. With no time-bound set, both the patients could complete the tasks with their affected hands with a much higher duration, resulting in TCT values greater than 100% (Table 4). 

### 4.2. Smoothness of Trajectory

The zero value of the trajectory smoothness parameter value indicates a perfectly straight line. With the number of obstacles increased, the subject needed to deviate from the central path to avoid errors; hence, the smoothness parameter increased (Table 5). Although it was observed that subjects had a gradual increase in the mean TCT for the module 2 tasks, because of the reduced time-bound, the tendency to deviate from the central path decreased and the mean smoothness values were observed to be gradually reduced. The smoothness values for both the patients’ unaffected hands are objectively closer to those of the reference values. When the patients could not reach the target before time-bound expires, the smoothness values were increased due to the relatively lower distance covered with their affected hands. Without any time-bound, these values are further increased due to high task completion time. 

### 4.3. Relative % Error

With an increase in the number of obstacles, the subjects traveled more distance to avoid the collision with obstacles, causing a relative increase in the values of percentage relative errors (Table 6). When the patients could not reach the target before the time-bound expired, these values decreased due to the relatively lower distance covered with their affected hands. Without any time-bound, patients could reach the target, resulting in values of relative % error identical to those of the healthy individuals.

### 4.4. Trajectory Plots

Representative trajectory plots (Figure 7 and Figure S7) can play as evidence for clinicians to evaluate the patient’s performance subjectively and distinguish if the user was able to complete a specific task within a specified time. The qualitative trajectory for a specific task level of affected hands of both the patients, considering healthy subjects as a reference to differentiate the performance of patients, was observed to be incomplete, not smooth, or having a higher value on the time axis. 

The comparison of these outcome measures obtained from the healthy subjects and the patients could be clinically relevant, providing more insights to therapists about patients’ performance and to serve as a base for future clinical studies. For example, the high TCT and trajectory smoothness values might indicate that increased focus on these parameters can increase coordination. Similarly, relative % error might indicate that the patient requires more intensive practice to increase precision while performing tasks. Apart from the clinical scales used for examining the improvements caused by rehabilitation intervention, such task performance metrics can also be additionally used as task-specific tools for appropriately monitoring the response to any intervention. The performance metrics obtained from the patients are expected to get closer to the reference values after an effective rehabilitation intervention.

Functional Magnetic Resonance Imaging (fMRI)- and Transcranial Magnetic Stimulation (TMS)-based neurological studies have demonstrated that repetitive practice of the affected limb resulted in decreased ipsilateral cortical activation and increased contralateral activation [31,32]. As reported, this increased activation resulting from intensive practice could be either due to the effective synaptic potentiation or vicariation (migration of activation from contra to ipsilateral) of the loss of neural networks in the ipsilesional hemisphere [31,33]. Neuroplastic changes have been reported in recent studies after VR-based interventions [34,35]. The developed VR tasks have the potential to provide an enriched environment for intensive practice of the affected upper limb, which is expected to facilitate neuroplasticity when used as an intervention regime. 

One of the advantages of VR-assisted training is the ability to evaluate the quantitative performances of a patient with stroke, which makes it more advantageous for use in clinical settings. In recent years, home-based tele-rehabilitation has gained popularity as it provides flexibility in rehabilitation location and duration [36,37]. In addition, the use of communication technologies enables clinicians to monitor patients’ progress from a remote location. In such situations, the patients’ quantitative performance at different intervention periods plays a crucial role in treatment response monitoring, deriving conclusions, or customizing the appropriate difficulty level of tasks [38]. However, very few of the available studies identified quantitative performance measures specific to the VR tasks designed in order to differentiate a healthy subject and a patient’s behavior while executing the VR tasks [38,39]. In a study to evaluate the efficacy of a gesture tracking-based robotic exoskeleton system, the authors of Reference [39] used the smoothness of various upper limb joint motions obtained from Microsoft Kinect tracking hardware and 3D trajectory plots to represent the motion quality of healthy individuals. However, both studies did not include any patient group for comparison. The study conducted by Alamri et al. [15] reported the use of TCT for ten healthy subjects obtained from different exercising VR tasks. The study suggested that TCT can be used as a potential performance metric to differentiate the performance level of healthy subjects and patients with stroke. The results showed that TCT values from three consecutive trials for a particular subject for the same task were observed to be gradually reduced. However, the above study did not report any patient-specific data. Another study performed by Schuster-Amft et al. [16] has used different parameters, such as efficiency and performance difficulty, as their performance metrics. Adams et al. [17] have used task-specific performance metrics, such as time to complete the task, normalized speed, and movement arrest period ratio, to assess the performance of the subjects. A moderate correlation between these metrics and the time-based Wolf-Motor Function Test (WMFT) was obtained. In addition, considering the fact that there is a variation in logistics, patient cohorts, patient sample size, and type of hardware used to give VR therapy, any direct comparison is not possible. 

Through this research work, the performance measures tailored to the designed VR tasks which can quantify the behavior of healthy subjects and patients with stroke were defined and validated. The primary objective of the current study was to define and validate the quantitative performance metrics from the developed VR tasks specific to the distal upper extremities. Another rationale behind the study was to obtain the subjective feedback regarding how to improve the overall VR setup from both the healthy subjects and the stroke patients. Please note that this study holds its importance by providing key outcomes towards establishing the framework and clinical protocol for patients with stroke for the future clinical study. Such preliminary study suggesting initial evidence is missing in the current literature. This is one of the limitations that we are addressing through this research work. The outcomes and learnings of this study will be really helpful in improvising the future study. The subjective learnings gained from the healthy subjects and the patients, such as customizing the VR tasks as per the requirement of individuals and the explored facts, such as incorporating fixed grip action, and customizing the range of motion of the used joystick, will play a crucial role in designing a major clinical study with a larger number of stroke patients.

### 4.5. Specific Observations Regarding Patients

It was observed that both the patients could not complete the tasks involving button pressing actions with their affected hands. P2, having less spasticity (MAS = 1), could use his index fingers without any assistance after three initial task levels. P1 felt more exhaustion than P2, possibly because of having higher spasticity (MAS = 2) at the wrist joint. After performing the tasks involving only wrist extension, P2 found the grasp and release of the wrist quickly; the reason might be attributed to the involvement of distal upper extremities during the task session. After repeatedly voluntary effort, P1 was able to re-position his index finger without the assistance of his unaffected hand. The patients had faced issues while maintaining a fixed grip due to the close placement of obstacles in later task levels of CE 2, 3, 4, and 5. 

Basically, establishing and modulating the complexity of task levels should be based on individual requirements and the ability of the patients. VR tasks have been implemented in various ways, e.g., different joysticks, Microsoft Kinect gaming console [15,16,17,40], Nintendo Wii [18,19] and Sony PlayStation [20], external wearable hand gloves, such as CyberGlove [13] and RAPAEL smart glove [5,14]; however, such commercial gaming consoles are not specifically customized for rehabilitation purposes and may require patients to make undesirable and difficult movements while performing the tasks. We observed a critical requirement of joystick tool for rehabilitation of patients with stroke, which should be customizable according to individual patient’s need with a higher range of motion, speed, higher range of wrist extension for focusing on flexor hypertonia, and also customizable in terms of spasticity of individual patients, given the observation that patient having MAS = 2 had difficulty operating the joystick than the patient with MAS = 1. Further increase in complexity levels of the proposed intervention could be achieved by incorporating different degrees of thresholds to actuate the joystick movements, adding the static hold action to increase the grip strength, and changing the button placements for the comfortable thumb and index finger movements. Overall, the entire setup was found to be capable of providing an interactive, user-friendly, and motivating platform to practice without any boredom or compliance, which supports the rationale behind designing our VR tasks. These observations about patients are to be addressed in future studies to improve the effectiveness of VR setup. After clinical validation, this low-cost, affordable, customizable, and easy-to-use VR setup can be used as a rehabilitation tool for distal upper extremities in low- and middle-income countries have limited resources and much need of rehabilitation services.

#### Limitations

This study has some limitations. Collecting feedback from more patients from different stages of stroke recovery and having different degrees of spasticity might give a broader insight into the modifications to be implemented in the future. No clinical scales were administered to objectively measure the perceived cognitive load during the sessions. The tasks developed in this study were more similar to “driving games,” which are to be customized according to the patient’s needs in our future study. The VR setup used in this study was a non-immersive one; hence, the level of immersion was not measured. The joystick used in this study has several inherent limitations, such as range of motion and the fact that button functions are not customizable, which can be addressed in future studies. Owing to this limited range of motion, the used joystick might not be able to serve as an ideal tool for helping stroke patients to train proper hand movements. In addition, the sample size of the patients (*n* = 2) was very small, and, in most of the task levels, the patients could not complete the tasks within the specified time limit. 

## 5. Conclusions

In this work, a library of VR tasks was developed for the rehabilitation of distal upper joints. The preliminary study results obtained from the conducted usability study on forty healthy subjects and two patients with stroke are also presented. Task-specific performance measures that could be used as a performance metric have also been identified. The performance metrics of the healthy subjects are to be used as a standard reference against which the patients’ performance is to be compared quantitatively. These quantitative performance metrics, along with the recorded subjective experience, will serve as a base for designing the protocol and addressing the existing hardware limitations required for future clinical trials on patients with stroke to investigate the potential of the developed system as an intervention tool. 

## Figures and Tables

**Table 1:** Demographic details of the two patients participated in the study.

Patient ID	Age (Years)	Sex (M/F)	Stroke	SAH (L/R)	Chronicity (Months)	MAS	FMA-UE
P1	27	M	Middle Cerebral Artery Infarct	L	51	2 (wrist), 1+ (fingers)	45
P2	39	M	Gangliocapsular Bleed	R	07	1+ (wrist), 1 (fingers)	55

Note: SAH: Stroke affected hand, MAS: Modified Ashworth Scale, FMA-UE: Fugl-Mayer Assessment of Upper Extremity, MAS ranges from 0 to 4 having grades 0, 1, 1+, 2, 3 and 4.

**Table 2:** Description of task difficulty levels in IE.

IE and Type of Track Used	Levels	Time Limit Specified (s)	Description of Environment
Number of obstacles (Track 1) [Module 1]	L1 (Track 1a) L1 (Track 1b)	70 70	Increasing the number of obstacles in each task level, keeping the time limit the same. Track 1a width = 7 units and Track 1b width = 9 units
Time Limit (Track 1b) [Module 2]	L1–L4	50, 45, 40, 35	Decreasing time limit with keeping number of obstacles same
Track 2 [Module 3] Track 3 [Module 4] Track 4 [Module 5]	L1–L2 L1–L2 L1–L2	45 60 90	In L2, button pressing involved keeping obstacles same as L1
Cognition task (a) [Module 6]	L1–L6	70	To remember the button sequence instructed and use the same sequence in the task to remove obstacles
Cognition task (b) [Maze Track 1–3] [Module 7]	L1–L3	70	Different maze tracks in which the subject has to find the correct path to reach the target

Note: IE: Individual Environment, L1: Task level 1.

**Table 3:** Description of task difficulty levels in CE.

CE and Type of Track Used	Levels	Time Limit Specified (s)	Description of Environment
CE1 (Track 1)	L1–L3 (Track 1a) L1–L3 (Track 1b)	65, 60, 55 65, 60, 55	In T2 no of obstacles is more than T1; in T3, the number of obstacles is the same as T2
CE2 (Track 2) CE3 (Track 3) CE4 (Track 4)	L1–L2, L3–L6, L7–L9, L10–L12, L13–L15	40, 55, 45, 55, 45	The number of obstacles is successively increased from L1 to L2. The number of obstacles for L3–L9 is the same as L1, and L10–L15 is the same as L2. From L3–L15, button pressing actions in different combinations are involved.
CE5 (Maze)	L1–L2 for Maze tracks 1, 2, 3, respectively	70, 65	In L1, obstacles are introduced in the maze track used in IE; in L2, random button sequence pressing action is involved.

Note: CE: Combined Environment, L1: Task level 1; the concept of task designing of CE 3 and CE 4 tasks are the same as that of CE2 tasks, with a different time limit.

**Table 4:** The variation of TCT parameter with the task levels of both the environments designed on track 4.

	Time Taken to Complete the Task (%)																
Task Levels (Track 4)	IE-L1	IE-L2	CE-L1	CE-L2	CE-L3	CE-L4	CE-L5	CE-L6	CE-L7	CE-L8	CE-L9	CE-L10	CE-L11	CE-L12	CE-L13	CE-L14	CE-L15
(Reference)	76.9	83.2	69.2	77.1	67.8	71.2	72.0	72.5	80.0	81.4	82.7	83.6	81.7	84.4	91.2	90.0	93.4
P1: unaffected	63.3	75.1	77.5	84.3	82.3	80.4	81.9	76.0	90.5	92.4	93.7	87.7	77.9	87.1	98.8	94.9	98.0
P1: affected	55.6	96.0	70.9	92.7	100.1	100.1	100.1	100.1	100.1	100.1	100.1	100.1	100.1	100.1	100.1	100.1	100.1
P1: affected(open-time)	85.1	113.7	60.3	90.0	67.3	141.3	124.0	106.4	142.0	119.6	120.0	117.8	107.5	118.6	124.3	138.0	114.5
P2: unaffected	72.3	87.0	87.5	95.0	87.8	93.0	88.9	93.8	96.3	93.7	98.7	93.3	94.0	93.0	92.6	96.2	97.7
P2: affected	81.1	100.0	83.4	100.1	100.1	100.1	100.1	100.1	100.1	100.1	100.1	100.1	100.1	100.1	100.1	100.1	100.1
P2: affected(open-time)	88.4	95.6	96.2	119.8	121.8	148.8	143.4	162.2	190.4	170.8	180.6	153.0	145.8	140.7	166.6	160.9	185.8

Note: Reference: Mean values of 40 healthy subjects, IE: Individual Environment, CE: Combined Environment.

**Table 5:** The variation of trajectory smoothness parameter with the task levels of both the environments designed on track 4.

	Trajectory Smoothness Values																
Task Levels (Track 4)	IE-L1	IE-L2	CE-L1	CE-L2	CE-L3	CE-L4	CE-L5	CE-L6	CE-L7	CE-L8	CE-L9	CE-L10	CE-L11	CE-L12	CE-L13	CE-L14	CE-L15
(Reference)	830	996	590	693	606	659	758	757	744	810	896	1073	1034	1046	1064	1071	910
P1: unaffected	613	691	494	610	606	654	721	336	825	818	1030	989	640	779	872	768	644
P1: affected	460	2780	423	1083	2329	4611	3456	3579	3328	3711	2966	2379	2637	3109	3127	3009	2709
P1: affected(open-time)	867	1856	405	1378	1009	6370	6814	7926	7408	8014	8106	7539	6814	6205	6726	6820	3987
P2: unaffected	687	1190	511	706	644	598	744	856	943	877	964	1002	784	746	837	779	725
P2: affected	712	1433	1419	3408	4700	3678	4109	3611	3329	4187	2278	2468	2258	2673	2648	3018	2308
P2: affected(open-time)	756	2257	573	1244	1632	3408	4425	5001	5358	6338	7002	5299	6814	7119	5668	6663	5887

Note: Reference: Mean values of 40 healthy subjects, IE: Individual Environment, CE: Combined Environment.

**Table 6:** The variation of relative % error parameter with the task levels of both the environments designed on track 4.

	Relative % Error Values																
Task Levels (Track 4)	IE-L1	IE-L2	CE-L1	CE-L2	CE-L3	CE-L4	CE-L5	CE-L6	CE-L7	CE-L8	CE-L9	CE-L10	CE-L11	CE-L12	CE-L13	CE-L14	CE-L15
(Reference)	−5.7	−5.3	−7.6	−7.6	−5.7	−6.9	−8.2	−8.9	−8.4	−8.3	−8.9	−7.4	−6.8	−6.9	−7.0	−6.8	−6.6
P1: unaffected	−14.4	−11.1	−8.6	−7.5	1.2	0.4	−3.2	−11.6	−8.4	−12.5	−10.3	−7.5	−12.2	−2.3	−8.7	−0.6	−11.0
P1: affected	−13	−19	−8.4	−4.7	−21.5	−57.5	−46.2	−42.1	−43.0	−51.2	−50.9	−45.2	−47.9	−48.3	−55.0	−52.0	−49.7
P1: affected(open-time)	−10	−13	−12.9	−11.6	−11.6	−9.4	−8.1	−11.0	−9.3	−10.3	−9.7	−6.3	−7.1	−4.1	−6.3	−5.2	−6.7
P2: unaffected	−8.7	−3.7	−5.6	−9.5	−3.2	3.4	−1.2	−8.6	−6.7	−10.1	−8.3	−6.5	−9.2	−8.4	−7.9	−7.1	−4.1
P2: affected	−9.2	−42	−31.3	−70.5	−32.5	−50.5	−59.2	−49.0	−63.0	−61.7	−57.9	−48.2	−61.0	−69.0	−55.8	−50.0	−45.7
P2: affected(open-time)	−6.7	−9.4	−2.0	−10.2	−9.3	−7.0	−11.7	−9.0	−5.0	−7.0	−8.6	−8.7	−6.5	−7.8	−6.9	−4.7	−3.7

Note: Reference: Mean values of 40 healthy subjects, IE: Individual Environment, CE: Combined Environment.

## Data Availability

The datasets used in this study are available from the corresponding author on reasonable request.

## Supplementary Materials

The following supporting information can be downloaded at: https://www.mdpi.com/article/10.3390/ijerph19031442/s1, Figure S1: showing the variation of TCT parameter with increase in difficulty levels in (a) Module 1a, (b) Module 1b, (c) Module 2, (d) Module 3, (e) Module 4, (f) Module 6, (g) Module 7: track1, (h) Module 7: track2, and (i) Module 7: track 3; Figure S2: shows the variation of TCT with the task levels of both the environments designed on track 4; Figure S3: showing the variation of Trajectory smoothness parameter with increase in difficulty levels in (a) Module 1a, (b) Module 1b, (c) Module 2, (d) Module 3, (e) Module 4, (f) Module 6, (g) Module 7: track1, (h) Module 7: track2, and (i) Module 7: track 3; Figure S4: shows the variation of smoothness values with the task levels of the environments designed on track 4; Figure S5. showing the variation of Relative % error parameter with increase in difficulty levels in (a) Module 1a, (b) Module 1b, (c) Module 2, (d) Module 3, (e) Module 4, (f) Module 6, (g) Module 7: track1, (h) Module 7: track2, and (i) Module 7: track 3; Figure S6: shows the variation of the percentage relative error values with the task levels of the environments designed on track 4; Figure S7: shows the trajectories of four representative healthy subjects (H5, H11, H14 and H20, and two patients with stroke for the task level CE3-L11.); Table S1: Subjective questionnaire feedback (SQF).
