# Supplementary material for: Design and Validation of Virtual Reality Task for Neuro-Rehabilitation of Distal Upper Extremities
Source: Int J Environ Res Public Health. 2022 Jan 27;19(3):1442. doi: 10.3390/ijerph19031442 (PMC8835157; doi:10.3390/ijerph19031442)
Supplement: Supplementary file 1 [file ijerph-19-01442-s001.zip › ijerph-1529836-supplementary.pdf]

Design and validation of Virtual Reality task for Neuro-rehabilitation of distal upper extremities

Supplementary Material

A library of Virtual Reality (VR) tasks was developed in this study and pilot-tested on forty healthy subjects and two patients with stroke. Three task-specific performance outcome measures- Time taken to complete the task (TCT), smoothness of trajectory, and relative % error was defined and computed for each of the task levels. Qualitative trajectory plots were presented for the subjective performance evaluation.

1. **Results of the calculated task-specific performance measures:**

   1.1 Time taken to complete the task (TCT)

   The variations in TCT values with various task difficulty levels in other tracks were shown in Figure S1. TCT values obtained from forty healthy subjects were treated as reference values and represented as box-whisker plot in the form of mean ± 95% confidence interval. TCT values obtained from the unaffected hands and the affected hands (both for time-bound and time-unbound conditions) of both the patients were plotted against these reference values for all the tracks. The mean TCT values of the healthy subjects were found to be increased with increase in task difficulty. It is clearly seen that the TCT values obtained from the unaffected hands (e.g., for CE2-L11: P1 = 87.2%, P2 = 92.8%, for CE3-L7: P1 = 92.7%, P2 = 90.9%, for CE5-L1: P1 = 87.3%, P2 = 94.5%, for Module 6-L2: P1 = 78.9%, P2 = 80.9%) were numerically closer to the mean TCT values (e.g., for CE2-L11: Reference = 83.8%, for CE3-L7: Reference = 89.1%, for CE5-L1: Reference = 85.2%, for Module 6-L2: Reference = 64.7%) obtained from the healthy subjects. TCT values obtained from the affected hands of both the patients (e.g., for CE2-L11: P1 = 100.1%, P2 = 100.1%, for CE3-L7: P1 = 100%, P2 = 100.1%, for CE5-L1: P1 = 100.1%, P2 = 100%, for Module 6-L2: P1 = 100%, P2 = 100%) were found to be higher than the maximum value of 100% in most of the task levels (Figure S1). For the affected hands of both the patients in time-unbound conditions, TCT values further exceeded the maximum value of 100% (for CE2-L11: P1 = 271.6%, P2 = 190.6%, for CE3-L7: P1 = 198.7%, P2 = 215.8%, for CE5-L1: P1 = 134.9%, P2 = 127.6%, for Module 6-L2: P1 = 166.1%, P2 = 138.1%).

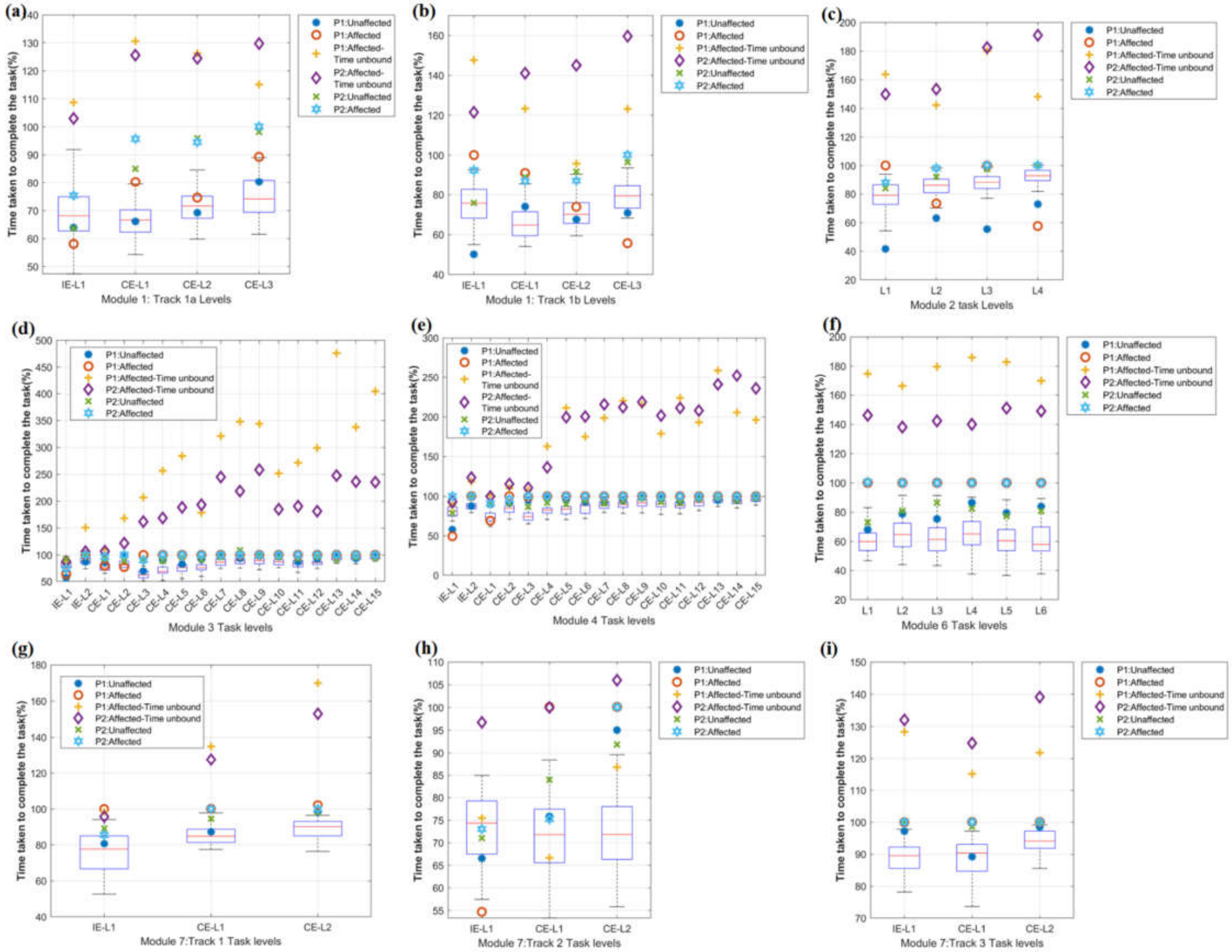

Figure S1. showing the variation of TCT parameter with increase in difficulty levels in (a) Module 1a, (b) Module 1b, (c) Module 2, (d) Module 3, (e) Module 4, (f) Module 6, (g) Module 7: track1, (h) Module 7: track2, and (i) Module 7: track 3. In each case, the scores of both the patients are plotted against the score of forty healthy subjects represented as the box-whisker plot in the form of Mean ± 95% C.I., which is taken as a reference. In most of the cases, TCT scores obtained from the affected hands of both the patients are equal to 100%. For the affected hands of both the patients in time-unbound conditions, TCT values further exceeded the maximum value of 100%.

During the 90 minutes' session, the mean TCT values of the healthy subjects increased relatively by 8.12% from IE-L1 (76.9 s) to IE-L2 (83.2 s) of the representative track 4 (Figure S2). Similarly, a relative increase of 34.9 % TCT was observed from CE-L1 (69.2 s) to CE-L15 (93.4 s) for track 4. However, a relatively decrease in the values of mean TCT have been observed from CE-L2 (77.1 s) to CE-L3 (67.8 s), CE-L10 (83.6 s), to CE-L11 (81.7 s), and CE-L13 (91.2 s) to CE-L14 (90 s), respectively.

Similarly, the unaffected hands of both the patients showed an increasing trend of TCT for IE (P1: 18.6%, P2: 20.3%) and CE (P1: 26.4%, P2: 11.6%) tasks of track 4 (Figure S2). For the affected hands of both the patients with time-bound included, a relative increase in TCT was observed in IE task levels (P1: 72.4%, P2: 23.4%). However, from CE-L3 to CE-L15, both the patients showed TCT values equal to 100% (Figure S2). For TCT values obtained from both the patients' unaffected hands without time-bound, relatively increased trends are observed for IE (P1: 33.6%, P2: 8.1%) and CE (P1: 89.8%, P2: 93.1% tasks of track 4. However, from CE-L3 to CE-L15, both the patients showed TCT values equal to greater than 100% (Figure S2).

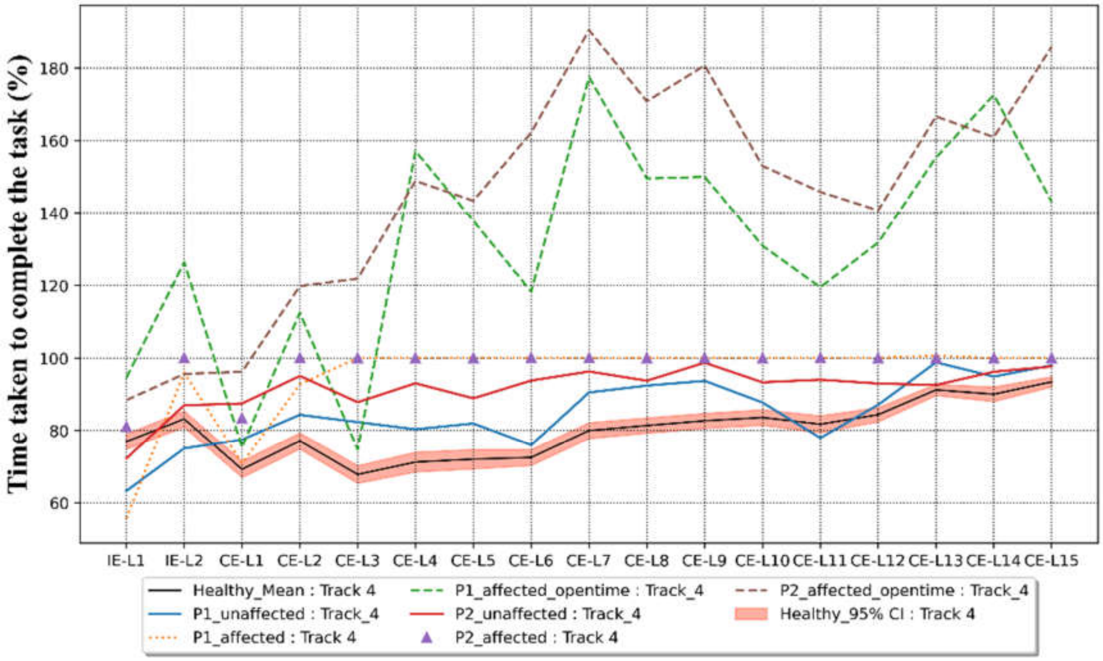

Figure S2. shows the variation of TCT with the task levels of both the environments designed on track 4. The mean values with 95% confidence intervals of healthy subjects have been plotted. Performance values obtained from unaffected hands of both the patients are almost identical to that of the healthy subjects, and higher values indicating the TCT values obtained from the affected hands of both the patients with and without given time constraints.

1.2 Smoothness of trajectory

The variations in trajectory smoothness values with various task difficulty levels in other tracks were shown in Figure S3. TCT values obtained from forty healthy subjects were treated as reference values and represented as box-whisker plot in the form of mean ± 95% confidence interval. Trajectory smoothness values obtained from the unaffected hands and the affected hands (both for time-bound and time-unbound conditions) of both the patients were plotted against these reference values for all the tracks. The mean trajectory smoothness values of the healthy subjects were found to be increased with increase in task difficulty. However, for module 2 tasks, it is found that trajectory smoothness values decreased with progression in task levels (Figure S2c). It can be observed that the trajectory smoothness values obtained from the unaffected hands (e.g., for CE2-L11: P1 = 513, P2 = 430, for CE3-L7: P1 = 461.8, P2 = 677.7, for CE5-L2: P1 = 832.4, P2 = 786.3, for Module 6-L2: P1 = 304.2, P2 = 255.1) were numerically closer to the mean smoothness values (e.g., for CE2-L11: Reference = 423.8, for CE3-L7: Reference = 497.2, for CE5-L2: Reference = 787.3, for Module 6-L2: Reference = 323.7) obtained from the healthy subjects. Trajectory smoothness values of the affected hands of both the patients (e.g., for CE2-L11: P1 = 1951.1, P2 = 1085.0, for CE3-L7: P1 = 1245.3, P2 = 1366.6, for CE5-L2: P1 = 1958.6, P2 = 1471.4, for Module 6-L2: P1 = 785.5, P2 = 743.3) are found to be considerably higher than the reference values (Figure S2). Trajectory smoothness values further exceeded (for CE2-L11: P1 = 10236.3, P2 = 2556.6, for CE3-L7: P1 = 7143.2, P2 = 8346.7, for CE5-L2: P1 = 5853.3, P2 = 1507.1, for Module 6-L2: P1 = 1002.5, P2 = 944.2) for the affected hands of both the patients in time-unbound conditions.

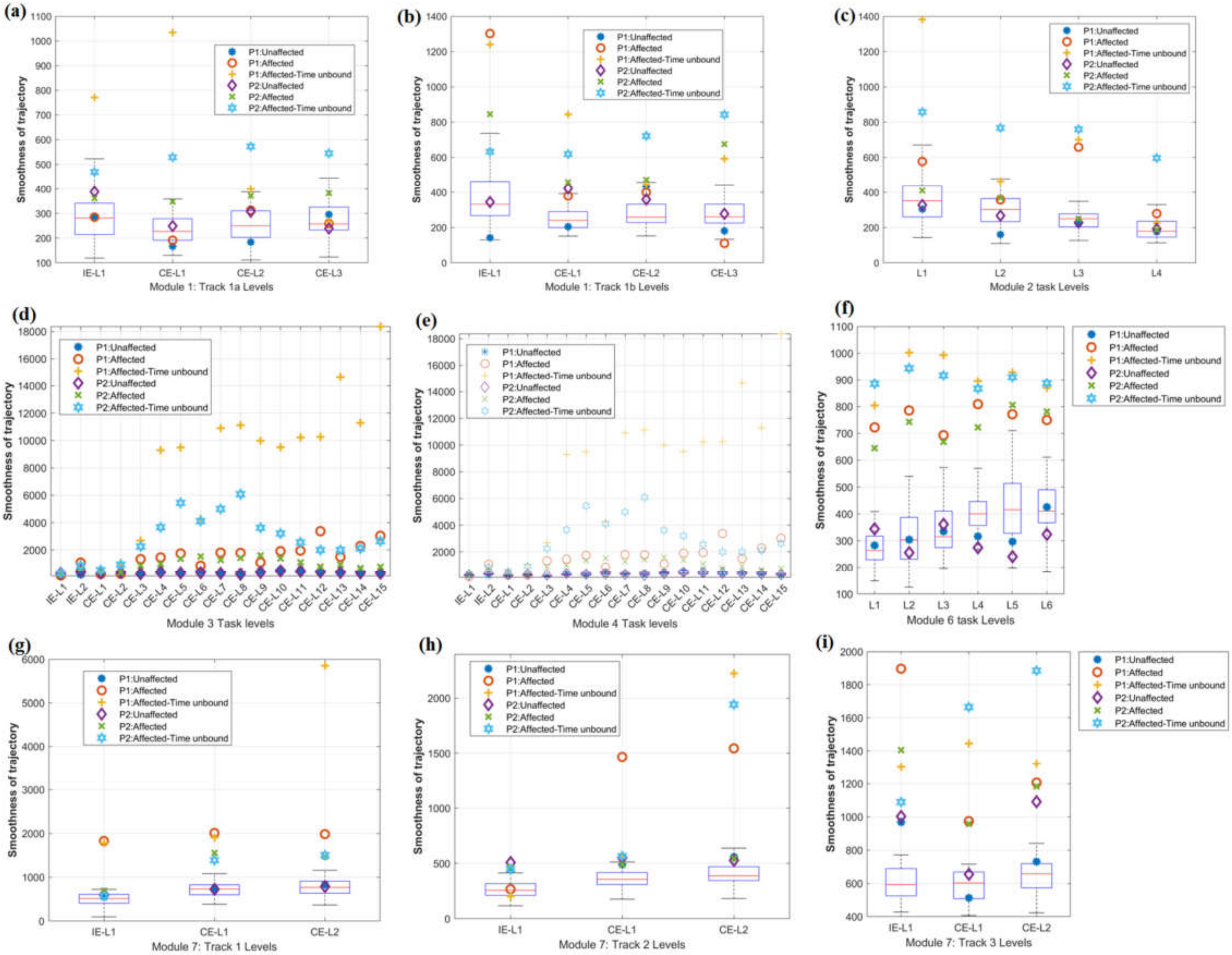

Figure S3. showing the variation of Trajectory smoothness parameter with increase in difficulty levels in (a) Module 1a, (b) Module 1b, (c) Module 2, (d) Module 3, (e) Module 4, (f) Module 6, (g) Module 7: track1, (h) Module 7: track2, and (i) Module 7: track 3. For each case, the scores of both the patients are plotted against the score of forty healthy subjects represented as the box-whisker plot in the form of Mean±95% C.I., which is taken as a reference. In most of the cases, Trajectory smoothness values obtained from the affected hands of both the patients are relatively greater than these reference values. For time-unbound conditions, trajectory smoothness values obtained from the affected hands of both the patients further increased.

The mean smoothness values of the healthy subjects increased relatively by 19.92% from IE-L1 (830.8) to IE-L2 (995.9) of the representative track 4 (Figure S4) during the task sessions. Similarly, a relative increase of 54.28% smoothness was observed from CE-L1 (590.1) to CE-L15 (910.3) for track 4. Similarly, the unaffected hands of both the patients showed an increasing trend of smoothness for IE (P1:12.7%, P2: 73.1%and CE (P1:30.2%, P2:41.9%) tasks of track 4 (Figure S4).

For the affected hands of both the patients with time-bound included, a relative increase in smoothness was observed in IE (504.1%, P2= 540.3%) and CE (P1 = 101.23%, P2 = 62.66%) task levels of track 4 (Figure S4). For smoothness values obtained from both the patients' unaffected hands without time-bound, relatively increasing trends are observed for IE (P1: 114%, P2: 198%) and CE (P1: 884.1%, P2: 926.4%) tasks of track 4 (Figure S4).

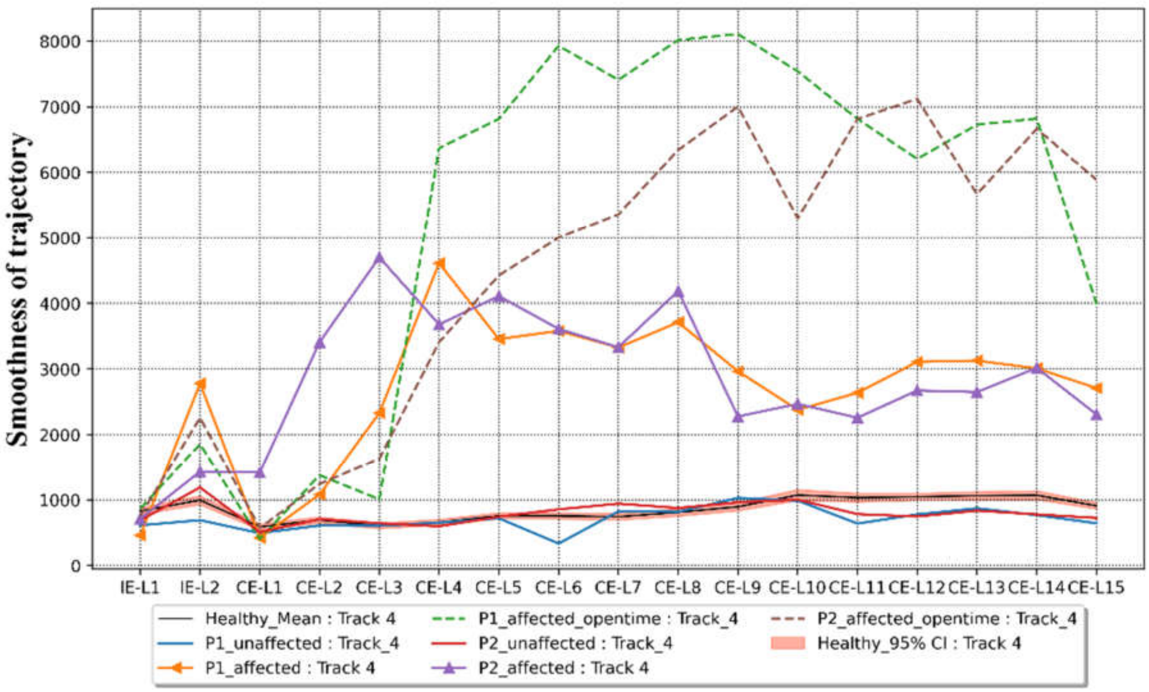

Figure S4: shows the variation of smoothness values with the task levels of the environments designed on track 4. The mean values with 95% confidence intervals of healthy subjects have been plotted. Performance values obtained from unaffected hands of both the patients are almost identical to that of the healthy subjects, and higher values indicating the smoothness values obtained from the affected hands of both the patients with and without given time constraints.

1.3 Relative % error

The variations in relative % error values with various task difficulty levels in other tracks were shown in Figure S5. Relative % error values obtained from forty healthy subjects were treated as reference values and represented as box-whisker plot in the form of mean ± 95% confidence interval. Relative % error values obtained from the unaffected hands and the affected hands (both for time-bound and time-unbound conditions) of both the patients were plotted against these reference values for all the tracks. It can be seen that the relative % error values obtained from the unaffected hands (e.g., for CE2-L11: P1 = 4.6%, P2 = 2.4%, for CE3-L7: P1 = 1.1%, P2 = 3%, for CE5-L2: P1 = −5%, P2 = 3.8%, for Module 6-L2: P1 = 3.6%, P2 = 3.6%) are numerically closer to the mean TCT values (e.g., for CE2-L11: Reference = 5.9%, for CE3-L7: Reference = 1.2%, for CE5-L2: Reference = 1.9%, for Module 6-L2: Reference = 4.2%) obtained from the healthy subjects. Relative % error values of the affected hands of both the patients (e.g., for CE2-L11: P1 = −58.7%, P2 = −57%, for CE3-L7: P1 = −56.2%, P2 = −60.3%, for CE5-L2: P1 = −27.9%, P2 = −18.2%, for Module 6-L2: P1 = −19.6%, P2 = −14.9%) are found to be considerably lower than the reference values (Figure S3). However, relative % error values obtained from the affected hands of both the patients (e.g., for CE2-L11: P1 = 5.6%, P2 = 3.7%, for CE3-L7: P1 = 8.6%, P2 = 4.1%, for CE5-L2: P1 = 9.3%, P2 = 4.3%, for Module 6-L2: P1 = 4.1%, P2 = 3%) are found to be numerically closer to the reference values obtained from healthy subjects.

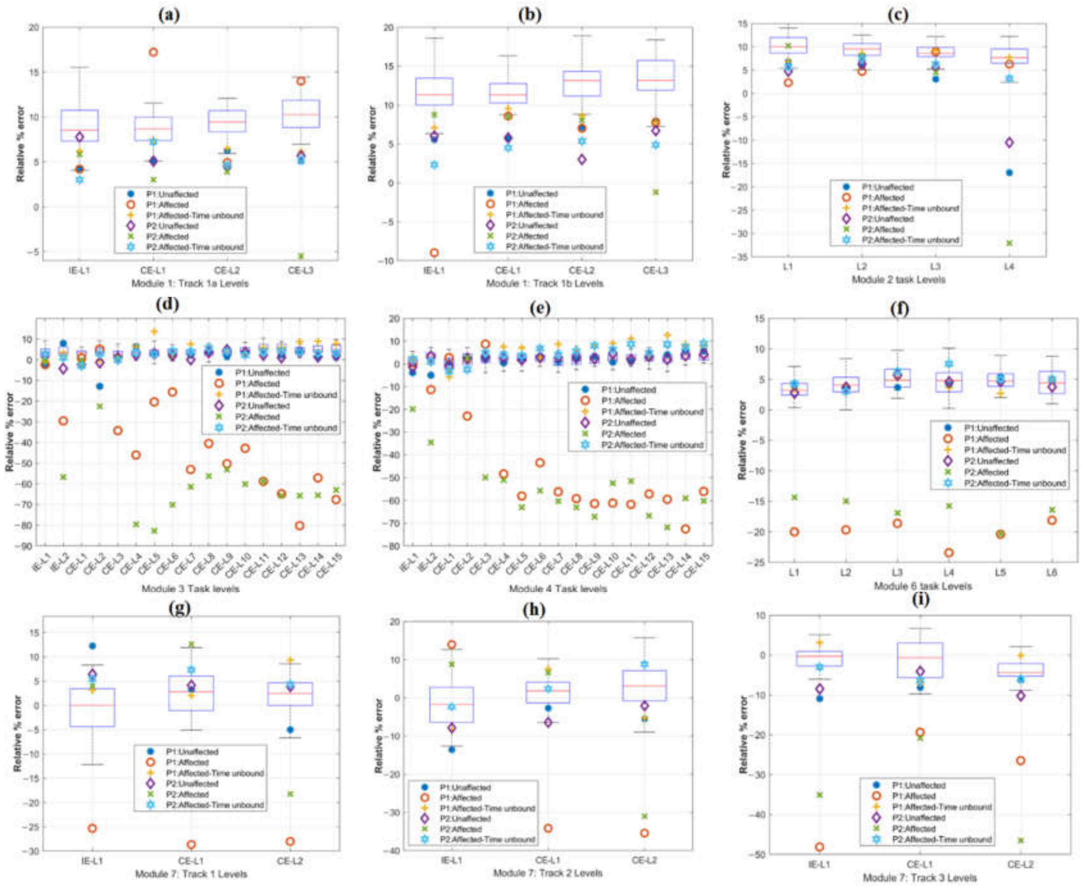

Figure S5. showing the variation of Relative % error parameter with increase in difficulty levels in (a) Module 1a, (b) Module 1b, (c) Module 2, (d) Module 3, (e) Module 4, (f) Module 6, (g) Module 7: track1, (h) Module 7: track2, and (i) Module 7: track 3. In each case, the scores of both the patients are plotted against the score of forty healthy subjects represented as the box-whisker plot in the form of Mean ± 95% C.I., which is taken as a reference. In most of the cases, relative % error values obtained from the affected hands of both the patients are relatively less than these reference values. For time-unbound conditions, relative % error values obtained from the affected hands of both the patients were found to be numerically closer to the reference values obtained from the healthy subjects.

The healthy participants' mean percentage relative error values increased relatively in IE (−5.7448 to −5.3559) and CE (−7.6258 to −6.6779) task levels of track 4 (Figure S6). Similarly, relatively increasing (P1: −14.4 to −11.1; P2: −8.7 to −3.7) values of relative % errors were observed while the patients performed the IE tasks using their unaffected hands (Figure S6). However, relatively decreasing (P1: −13.7 to −19.6; P2: −9.2 to −42.7) values of relative % errors were observed while the patients were performing the IE tasks using their affected hands with time-bound given (Figure S6). Relatively decreasing (P1: −10.7 to −13.4; P2: −6.7 to −9.4) values of relative % errors were observed while the patients performed the IE tasks using their affected hands without specifying time-bound (Figure S6).

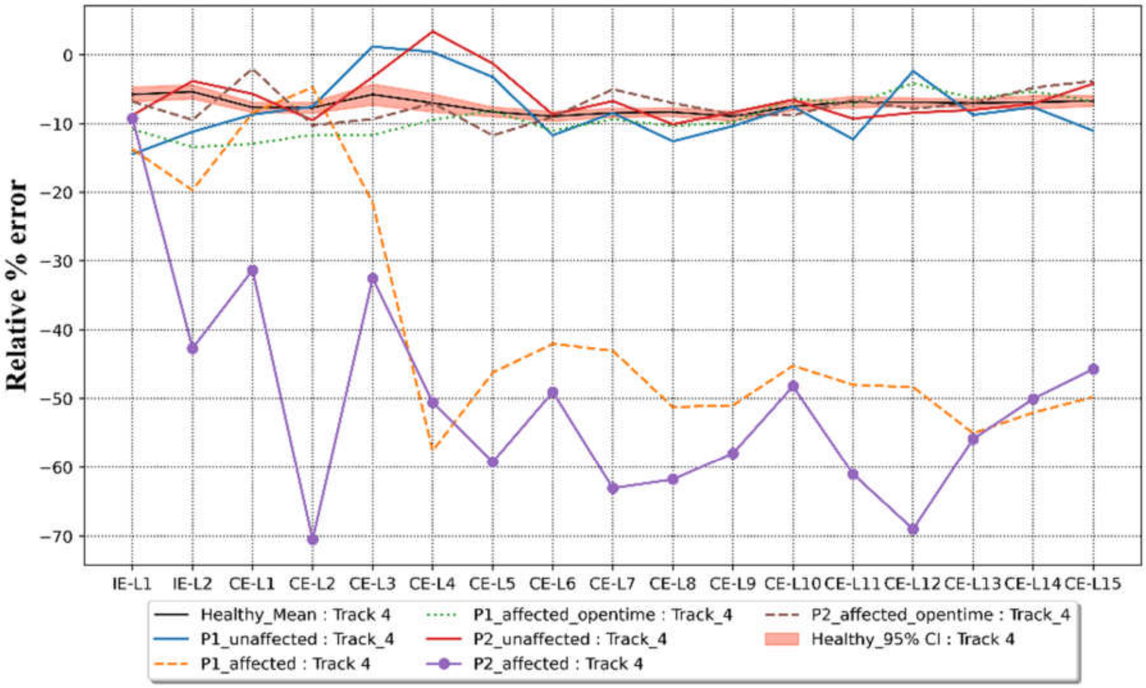

Figure S6 shows the variation of the percentage relative error values with the task levels of the environments designed on track 4. The mean values with 95% confidence intervals of healthy subjects have been plotted. Performance values obtained from unaffected hands of both the patients are almost identical to that of the healthy subjects, and lower values indicating the smoothness values obtained from the affected hands of both the patients with and without given time constraints.

1.4 Trajectory plot:

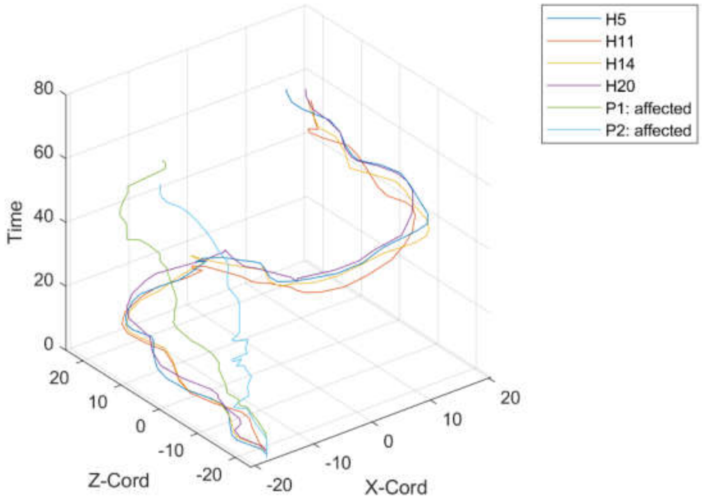

Figure S7 showing the trajectories of four representative healthy subjects (H5, H11, H14 and H20) and two patients with stroke for the task level CE3-L11.

2. Subjective Questionnaire Feedback for healthy subjects and patients:

A self-designed subjective questionnaire form (SQF) has been administered to record healthy subjects' and patients' experience regarding the usability of the joystick and VR tasks (Table S1). The SQF focused on the subject's interestingness, and controllability of the VR tasks, user-friendliness, pain/fatigue/ adverse event experienced, individual suggestions, etc. The SQF has two parts: part A is common to both healthy subjects and patients; part B is specific to patients.

2.1 Results Summary of SQF

All the healthy subjects and the patients found the entire VR setup safe and easy to use even at home, provided with the details of instructions and proper demonstration before using the system. All of them found the VR tasks easily understandable, enjoyable, motivating, and exciting to perform. Suggestions obtained from both healthy subjects and patients were noted focusing on how to improve and customize this VR setup further. The details of SQF form and obtained feedback are given in Table S1.

Table S1. Subjective questionnaire feedback (SQF)

**Part A: Common questions to both healthy subjects and patient**						
**No.**	**Questions**	**(%) healthy subjects said Yes**	**(%) healthy subjects said No**	**Comments by healthy subjects**	**Patients said Yes / No**	**Comments by Patient**
1	Were the tasks simple and interactive?	100	0	-	Yes (P1, P2)	-

2	Were you able to understand the procedure and synchronize with it clearly?	85	15	Slight problem in understanding the procedure at initial levels, as task levels goes on the issue solved	Yes (P1, P2)	-
3	Did you experience any tiredness or fatigue during the protocol duration?	45	55	Yes, due to continuously performing tasks, needed rest period in between task levels	Yes (P1)	I need rest in between task levels (P1)
4	Do you find the joystick easy and comfortable to operate after trial session?	80	20	Needed one or two more trials to be comfortable	Yes (P1, P2)	Yes, but found difficulty in operating buttons
5	Did you face any difficulties while understanding the task instructions?	22.5	77.5	It took relatively more time to understand the cognitive task instructions	No (P1, P2)	-
6	Did you find the tasks interesting, engaging and motivating?	100	0	-	Yes (P1, P2)	-
7	Was the task GUI interesting?	100	0	-	Yes (P1, P2)	Top view pane size to be increased for a better visibility
8	Is the goal of task clear, engaging and interesting?	100	0	Yes, each level of tasks has different goals to execute and clearly explained before the tasks.	Yes (P1, P2)	-
9	Do you want to perform the tasks again?	85	15	Repeating same tasks is a bit boring. I don't want to do it today, but might be interested later.	Yes (P1, P2)	Yes, I want to execute the tasks at home again.
10	Are the text instructions, buttons and time counter	100	0	-	Yes (P1, P2)	-

	clear and readable?					
11	Does the lag between real and virtual movements acceptable?	100	0	No lag experienced	Yes (P1, P2)	No lag experienced
12	Any visual discomfort perceived during session?	35	65	Especially occurred in later tasks of combined environment tasks and cognitive remembering tasks. Slight headache after executing tasks for long time.	Yes (P1)<br><br>No (P2)	Experienced during CE tasks
13	The rest between the task levels is fine?	60	40	initial task levels don't require much rest in between. More rest period is needed in CE tasks (level 10 onwards) and cognitive tasks.	Yes (P1, P2)	-
14	Any postural fatigue (excluding arm) felt?	35	65	Continuous handling joystick and performing causes minor back and shoulder pain.	Yes (P1)<br><br>No (P2)	At one-third dorsal position of forearm (P1)
15	Do you find the difficulty levels too difficult to execute?	10	90	Due to less time limit could not able to complete some CE tasks at first attempt, but able to do in next attempt.	No (P1, P2)	Difficulty faced in button pressing tasks and not able to complete in time.<br>CE tasks were relatively difficult.
16	Do you find the cognitive tasks too difficult to execute?	0	100	-	No (P1, P2)	-
17	Was controlling the virtual movements with joystick easy?	100	0	-	Yes (P1, P2)	-

18	Any adverse event experienced?	0	100	-	No (P1, P2)	-
19	Do you feel it safe to use?	100	0	-	Yes (P1, P2)	-
20	Did trajectory thickness and the panel size allow good playability of the game?	100	0	-	-	Trajectory thickness in CE 4 & 6 tasks to be increased (P1)
21	Were you able to use the wrist and fingers as promptly as required?	95	5	-	Yes (P1, P2)	Difficulty faced in placing fingers perfectly
22	How many VR sessions / tasks or how much duration it took to be comfortable?	-	-	After playing 4–5 task levels (5 minutes appx.) the sessions became comfortable	-	No problem faced after the demonstration and trial tasks (P1, P2)
23	Was the audio-visual feedback useful and interesting?	100	0	More interesting & different sounds and graphics could be added for future	Yes (P1, P2)	-
24	Do you feel your attempt is proportional to the task executed in going straight, back, taking left and right turns?	100	0	-	Yes (P1, P2)	-
25	Is the duration of the tasks fine – long /short?	70	30	-	-	Long (P1) Fine (P2)
26	Do you think you can use the setup at home?	100	0	Yes, provided well demonstration and instructions on how to use	Yes (P1, P2)	I will prefer to use this setup at my home at my own convenience as it is easy to

						understand and use.
27	What did you like in the VR sessions?	-	-	A first-time and new experience of playing games that can give a feel of real -world objects<br><br>Different from as usual mobile/pc games<br><br>Controlling through joystick was a nice experience<br><br>In each task something different to execute<br><br>GUI and 3d object interaction interesting	-	Interesting tasks to perform as compared to exercise tasks (P1)<br><br>Could perform tasks for a long period at a time without boredom (P2)
28	What did you dislike in the VR sessions?	-	-	Longer duration<br><br>Repeated use of same 3d objects<br><br>Postural pain after a long use	-	Longer duration (P1)<br><br>Use of similar environments and objects (P1, P2)<br><br>Visual fatigue due to using for a longer time (P1)<br><br>Sweating after long gripping of joystick (P1)<br><br>Joystick not that much comfortable to be used with left arm (P1)
29	Did you have VR exposure earlier?	0	100	-	No (P1, P2)	-

30	Is there anything you would like to change in the session?	-	-	To add new 3d objects and task environment<br><br>To add Interesting visual and audio cues<br><br>Increase the specified time limit for CE tasks<br><br>To reduce overall duration of tasks with rest period	-	Use of new 3d objects and audio-visuals (P1, P2)<br><br>Reduce overall duration of training (P1)<br><br>Increase the size of trajectory and top view window (P1)<br><br>Soft-cloth to be used instead of pillow for comfortable positioning of arm (P1)<br><br>Buttons of joystick to be placed at more comfortable position (P1, P2)

SQF part B: Questions specific to patients

No.	Question	Remarks
1	Do you prefer doing these virtual tasks over real-world exercise tasks?	Yes<br>(P1, P2)
2	Do you feel any improvement after involving your hand in the tasks?	Yes, I could use index finger without help of unaffected hand after voluntary effort and control the wrist movements smoothly (P1, P2)
3	Did you feel you could prevent the involvement of elbow and shoulder joints?	No<br>(P1, P2)
4	How satisfied you were with the treatment? (1–5)<br>[1-lowest score, 5-highest score]	5<br>(P1, P2)
5	Did you feel the hand stiffness increased during the attempt or the execution of the tasks?	Yes, in fingers<br>(P1, P2)

6	Did you feel you were able to control the joystick smoothly during the task or after few minutes in the tasks?	Yes, wrist extension was a little difficult (P1)<br>No (P2)
